# AlliumMap-A comparative genomics resource for cultivated *Allium* vegetables

**DOI:** 10.1186/1471-2164-13-168

**Published:** 2012-05-04

**Authors:** John McCallum, Samantha Baldwin, Masayoshi Shigyo, Yanbo Deng, Sjaak van Heusden, Meeghan Pither-Joyce, Fernand Kenel

**Affiliations:** 1 The New Zealand Institute for Plant & Food Research Ltd, Christchurch, Private Bag 4704, New Zealand; 2 Department of Biological and Environmental Sciences, Faculty of Agriculture, Yamaguchi University, 1677-1 Yoshida, Yamaguchi-shi, Yamaguchi 753-8515, Japan; 3 Applied Computing Group, Faculty of Environment, Society and Design, PO Box 84, Lincoln University, Lincoln, 7647, New Zealand; 4 Wageningen University and Research Centre, Postbus 16, 6700 AA, Wageningen, Netherlands

## Abstract

**Background:**

Vegetables of the genus *Allium* are widely consumed but remain poorly understood genetically. Genetic mapping has been conducted in intraspecific crosses of onion (*Allium cepa* L.), *A. fistulosum* and interspecific crosses between *A. roylei* and these two species, but it has not been possible to access genetic maps and underlying data from these studies easily.

**Description:**

An online comparative genomics database, AlliumMap, has been developed based on the GMOD CMap tool at http://alliumgenetics.org. It has been populated with curated data linking genetic maps with underlying markers and sequence data from multiple studies. It includes data from multiple onion mapping populations as well as the most closely related species *A. roylei* and *A. fistulosum*. Further onion EST-derived markers were evaluated in the *A. cepa* x *A. roylei* interspecific population, enabling merging of the AFLP-based maps. In addition, data concerning markers assigned in multiple studies to the *Allium* physical map using *A. cepa*-*A. fistulosum* alien monosomic addition lines have been compiled. The compiled data reveal extensive synteny between onion and *A. fistulosum*.

**Conclusions:**

The database provides the first online resource providing genetic map and marker data from multiple *Allium* species and populations. The additional markers placed on the interspecific *Allium* map confirm the value of *A. roylei* as a valuable bridge between the genetics of onion and *A. fistulosum* and as a means to conduct efficient mapping of expressed sequence markers in *Allium*. The data presented suggest that comparative approaches will be valuable for genetic and genomic studies of onion and *A. fistulosum*. This online resource will provide a valuable means to integrate genetic and sequence-based explorations of *Allium* genomes.

## Background

The large monocot genus *Allium* comprises hundreds of species and includes several with great economic, culinary and health value. Onion and shallot (*Allium cepa* L.; 2n = 2X = 16) are among the most economically significant monocot species outside the commelinoid grasses [1]. *A. fistulosum* (Japanese Bunching or Welsh Onion; 2n = 2X = 16), leek (*A. porrum*; (2n = 4X = 32) and garlic (*A. sativum*; 2n = 2X = 16) are widely grown and traded, with many other species being locally significant as spices and flavorings. *Allium* species are notable for their very large genomes, typically in the range 10–20 Gbp [2], which have complicated genomic studies and precluded genome sequencing to date. Genetic map development in onion and other *Allium* has been limited by difficulty in developing, maintaining and exchanging genetic stocks, high degrees of heterozygosity, and a dearth of sequence data [3].

The first published genetic map of an *Allium* species was that developed by King and colleagues [4] in the intraspecific onion cross 'BYG15-23 x AC43'. Constructed initially using RFLP markers, this map was subsequently augmented with SNP and SSR markers derived from EST sequencing [5,6]. These more portable markers enabled partial map construction in other intraspecific onion crosses to enable map-based genetic analysis of fertility restoration [7], color [8] and other bulb traits [9,10].

The breeding systems of *A. fistulosum* have facilitated development of several larger mapping pedigrees and detailed genetic maps based initially on SSR and AFLP markers [11,12]. These maps were used to conduct QTL analysis for seedling vigor [13]. More recently Tsukazaki and colleagues [14] reported a further *A. fistulosum* map based on *A. fistulosum* genomic SSR markers and onion EST-derived SNP and SSR markers, providing further scope for comparative studies between onion and *A. fistulosum* genomes. The only *Allium* relative known to readily produce fertile hybrids with onion is *A. roylei*[15], which has been used to develop an interspecific map [16] and backcross progenies with valuable disease resistance [17,18]. Since *A. roylei* also crosses with *A. fistulosum*, this has enabled development of bridge crosses containing all three genomes [19], thus enabling a potential path for introgression of *A. fistulosum* genetics into onion.

The key resource that has enabled alignment of *Allium* genetic maps to physical chromosomes and facilitated comparison among species is the sets of *A. fistulosumA. cepa* alien monosomic addition lines (AMALs) developed by Shigyo and colleagues [20]. These were initially applied to anchor AFLP-based maps in the interspecific *A. cepa* x *A. roylei* cross [21] and subsequently to anchor the 'BYG15-23 x AC43' map [6]. Subsequently they were used to anchor SSR-based maps in *A. fistulosum*[12] to physical chromosomes, and more recently to assign many more onion EST-derived anchor markers used in *A. fistulosum* maps [14].

In other studies, a large number of phenotypic and molecular markers, including many candidate genes relating to economic traits, have also been assigned to chromosomes [6,22-26], providing a valuable guide for functional and QTL studies. These findings have been reported in diverse publications but have not to date been available in an accessible or integrated manner.

Genome sequence, map and marker data from *Allium* species have to date been limited and difficult to access. Marker assays from the 'BYG15-23 x AC43' population have been accessible through Genbank [27] and garlic EST data have been presented through a web database [28]. Recently, Bhasi and colleagues [29] presented RobustDb, a generic online genomics database most notably containing garlic map and marker data. The VegMarks database [30] contains detailed information concerning *A. fistulosum* markers. Neither of these databases provides comparative data. Increasing development of doubled haploid stocks [31,32] and availability of next-generation sequencing mean that *Allium* marker and map resources will expand rapidly in the near future. Therefore it is important to provide existing map and marker data in an accessible form with links to underlying sequence, to enable integration of new data with past studies.

Comparative genomic approaches have been widely used and proven in crop genetics, and are of growing interest as improved sequencing technologies enable ever broader and more detailed surveys of germplasm [33]. Online databases integrating genetic map, marker, sequence and germplasm data such as Gramene [34] and GDR [35] are now key tools for publishing and exploiting such data from the monocot grasses and the Rosaceae family respectively. Given their economic significance, there is a clear and pressing need for such resources in *Allium*.

The use of many common onion EST-derived markers and the extensive use of AMALs to anchor both onion and *A. fistulosum* maps provide the potential for similar comparative approaches to be used in *Allium* genetics and genomics. In this study we present an integrated view of genetic maps in onion and *A. roylei* and an online database in which these can be explored.

## Construction and content

### Interspecific allium map integration

The interspecific *A. cepa* x *A. roylei* interspecific map was augmented with additional genetic markers to increase correspondences among *Allium* maps. A total of 107 markers comprising 73 additional onion EST-SSRs, 3 *A. fistulosum* genomic SSRs and 31 SNP markers derived from onion ESTs were evaluated in the population previously used to construct an AFLP-based linkage map [16] using previously published methods [9]. Previously unpublished markers are shown in Table 1. Revised genetic maps were calculated using JoinMap 4.0 software [36]. Linkage groups were first formed using LOD 5 cutoff from two data sets each containing co-dominant markers plus dominant markers from one parental phase. These were then merged and linkage maps constructed using default settings and Kosambi distances.

**Table 1:** **Previously unpublished primer sets mapped in the interspecific *Allium cepa *x *A. roylei *population**

**Primer Set**	**Genbank Accession**	**FORWARD PRIMER**	**REVERSE PRIMER**
ACABE58	CF447676	TCTTCGAGAACTATCCCGACAT	ACTCAACCGCTGTTACAAGGAT
ACI017	AY585678	CCGACTACATGTAAGTTGCATTAAC	TCTTGCATAATTTCACTGCACA
ACM005	BI095610	CGCTTCAGCAGTGAGTTGTT	TGTTGTCCGATACAGAGTTGCT
ACM021	CF448154	AAAACCCTCAACATCTCACTCC	TCTCTTCTTCCTCGTCCTGC
ACM037	CF438925	GACCGACTCCAAAGCCATA	CTCTCCCGTTCTCAAAATGC
ACM049	CF447728	TAACGACATCCCTACCGC	GCTTCTTCTTCCACTTTCGG
ACM050	CF447828	GGTTCTCTGTTTGGGACA	CCGTTTCGGCTACCTTGTAT
ACM052	CF441811	CAGCAGCAACAAAGAATGC	CTGGGGAGAATGAGAAGCAC
ACM053	CF437211	CTGGGCTCTTTTGTTCATCC	ATGGTGGAGGTATGTGAGGG
ACM058	CF435771	GGAGTCACACAACAGAAACACAA	AAGAAGGAATAGAGATGTAGCCGA
ACM060	CF435985	ATCAGCAGCCTTCCCAGTAA	ATCACACCCGCAAAAGAAT
ACM065	CF449328	GCTCTGATGGAGGATGGTTC	CTTGCCATCTTTGTCGGT
ACM072	CF441584	TGAATTCAGGCCAAACATGA	GAGGAAAGCCTGAAGAGTAGCA
ACM076	CF449018	ATTAGAAACATCCATCGCCG	CGCGATCATCATTTTCCATA
ACM080	CF449761	GCATTATGCAGTAACGGGCT	GCAGCAGCATTTGATTGAAC
ACM081	CF447998	CTGAAAAGAAACCCGCAGAG	TCAGGATGCACTTGCTTCAG
ACM082	CF436620	CACCGTTCCTCAGCTCACTT	AGAGGGACGAAATGAAAGCA
ACM092	CF451134	GTGATTTGAAGCCACCACCT	TGAATGGTGGTTATTCGGGT
ACM096	CF446191	TGTGGGCAATTCACGTTATG	AAAAGTTGTGAACGGCATCC
ACM105	CF441894	CAAGTGGAGCGGGTATTTGT	GAGGCACAACTTCCTCTTCG
ACM107	CF449837	CCTTCATTCCCAAAGCACAT	GCGATAAAGAGGGACAGCAG
ACM114	CF436720	TAAGTTTTGCCTCCACCACC	GCTCCACTTCAAGGCTGTTT
ACM129	CF442903	CTAGGTTTCCGTGCTCCAAG	CAGTTGGAGATCAACAGGCA
ACM140	CF442000	TTGAAGCTATTCTCCGCAGC	AGGGGGTCATTGATCCTAGC
ACM144	CF441789	GCAACGGTAGAAGAACCTGC	AACCTCTTTTGGTGCCTCCT
ACM149	CF440830	GAAGATGGGTTTGAGTGGGA	CAAGCCTGCCCTTACTCTTG
ACM174	CF451831	TGCCCAATTATCGTTTCCAT	GATGAGGCGAGTTTAGAGCG
ACM183	CF443106	GATGATGGTGATGGCATTGA	GTTTGCAGGCTCCATTGATT
ACM231	CF441488	AAAGCTTCTACCCTGGCGAT	TCCCTACGAACTCGTCATCC
ACM238	CF443464	TGATAGCCAGTTGATTGCGA	TTCCCCAGTACACACCTTCC
ACM240	CF444554	GTGCAACTCCAAGAGAAGGG	AATATAAAGGCGTTGGCCTG
ACM245	CF445289	GGATCTGATCGGAGATTGGA	GCGCACCTCTCTGCTAGACT
ACM255	CF449065	AAATTCCCAAAACGAAACCC	GGGTTTCAGGAACAGTCAGC
ACM295	CF445600	AGATCCGTCCCATGAAACT	GATCCGCTTCTGAAATCTCG
ACM304	DQ273270	GAATTTAGGCCCATTTCAAGG	TGATTTGCCTAATGTTTTTACG
ACM322	ES449660	TTCTTCTCCTATCCAGCTATCG	GTGATTTGGGAGGGGATTTT
ACM340	CF437547	AAGTCTGGTGGTTGGTCCAG	GGTGCCCAAGAAGTTGGTTA
ACP002	AA451591	AAGCTTCTTTCGATCCTTTGTG	GCTTCGATTCCATTTCAAGTTC
ACP003	BE205590	AAGCTCTTAAAGCTGCTGATGG	ATGCACGATAGCACAAGACATC
ACP034	BQ580357	CAGTCTTGTGGTCATTGGTCA	AACCCATGCGTATTTGAAGG
ACP052	CF445805	TTCCCTCCTCACTCCCTACA	CGACCACAAACACAAGCAAC
APSR	AF212155	CAGCTGCAGACTTTTCCTAC	CCACGTGATCGAGTAGATCGT
GGCS	AF401621	CTGGAGTCACACCTGCAGAG	TCGCCTTCGGAACTGTTATT
GGT	AF401622	TGTTGCTACCGATGATGGTC	ATGCAACCCTGCAATTTCTC
SPS3'UTR	EU164758	AAAGGGAGATACAGACCAT	ATTATACATCTCATCATGTCACT
SUCS	CF440928	TTTGAAGTGTGGCCTTACCTTGAG	TGATGAAGTCTGTTCGATCATGGC

### Database configuration and curation

Map and marker data provided by authors of previously published linkage mapping studies [4,8,12,14,16,37] were compiled in a MySQL relational database and reformatted in a form suitable for import into CMAP [38]. Marker data from the `BYG15-23 x AC43' cross [6] were reformatted in cross-pollinator format for JoinMap 4 and linkage maps were recalculated using default settings. Correspondences between loci with identical names were added using the cmap_admin.pl utility provided in CMap, or manually added based on use of common underlying sequences, as identified through information provided by authors and/or identified in the MySQL database. Further correspondences were identified by cross-checking primer sets against the Onion Gene Index [39] using the primersearch tool from the EMBOSS suite [40] and creating correspondences for any marker pairs amplifying the same sequence. AMAL data were compiled into a Google® spreadsheet and published in searchable form using Simile Widgets http://www.simile-widgets.org[41]. Sequences used for marker design were re-formatted to include marker names in fasta header lines and formatted to provide a BLAST [42] database. Information concerning PCR primer sets used to reveal SNP and SSR markers is provided via custom SQL queries to an external database included in modifications to the distributed CMAP feature information templates. QTL information was compiled from published data and manually added as map features using the CMAP administrator interface, or bulk uploads with the cmap_admin.pl tool.

## Utility

The resources provided at http://alliumgenetics.org may be browsed through direct links to maps organized by species and publication, or through the standard CMAP interface. Markers or any other features may be searched using the built-in feature search option in CMAP, or through a simple form interface provided to enable searching for details of specific markers or primer sets. A BLAST facility is provided to enable querying any sequences of interest against targets of existing markers.

The markers assigned using AMALs may be browsed and filtered through a web page and the RDF data source may be used as input for other Web2.0 mashups [43].

AlliumMap currently contains 1,776 markers from 10 *Allium* maps and 512 correspondences between markers. Genetic maps may be browsed through a standard CMAP interface, and marker hyperlinks provide access to marker information including links to GenBank sequences and other marker assay details.

## Discussion

### Integration of the interspecific *allium* map

**Figure 1:**
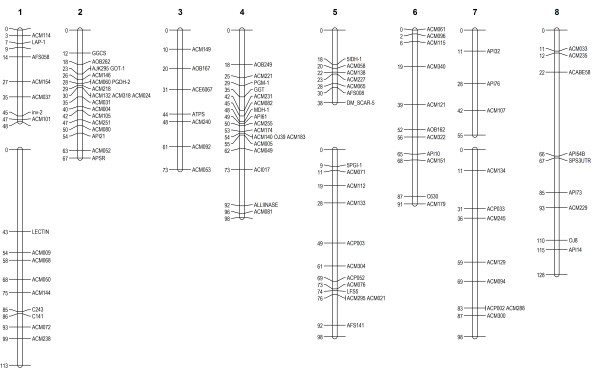
**Isozyme and PCR-based marker anchor loci on integrated *A. cepa* ‘Jumbo’ x *A. roylei* interspecific map.** Numerals at top denote chromosome number based on AMAL assignments. Scale denotes distance in Haldane units (cM). AFLP markers are omitted for clarity.

The addition of 74 co-dominant markers to the *A. cepa* x *A. roylei* interspecific map has enabled integration of male and female maps previously constructed primarily with dominant AFLP markers. The map comprises 11 linkage groups spanning 1 Morgan (Figure 1) compared with a length of 660 cM (Kosambi) reported observed for the original AFLP-based map [16]. This is the expected map length for onion based on chiasma frequency [44] and suggests that this map spans most of the genome. The combination of anchor loci assigned using AMALs and mapped in the interspecific cross has provided many additional landmarks for aligning genetic linkage maps in *A. cepa* and *A. fistulosum*. Alignment of linkage groups in this cross with the 'BYG15-23 x AC43' onion map [6] reveals useful synteny, as reported previously in studies of onion chromosome 8 [9].

Approximately 30 % of onion EST-derived PCR-based markers do not amplify in *A. roylei*, but may nevertheless be mapped as dominant markers in the *A. cepa* x *A. roylei* cross. This high degree of polymorphism means that this cross is extremely useful for developing detailed genetic maps. Development of additional crosses of this type for mapping with new SNP and other marker resources developed with next-generation sequencing in onion would be desirable to provide highly informative stocks for researchers mapping new genes of interest.

### Consensus maps in *allium*

**Figure 2:**
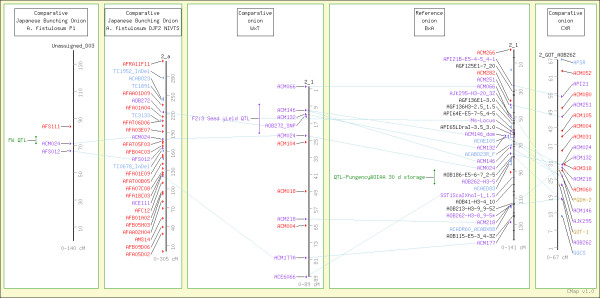
**AlliumMap comparative view of *Allium *chromosome 2, based on genetic maps from onion **[6]**,**[8]**,**[10] and ***A. fistulosum***[12].

The present database contains 512 correspondences between markers on different *Allium* maps. Map comparison reveals useful degrees of expressed marker portability and suggests considerable potential for comparative methods to resolve common questions of crop evolution, biological function and economic trait regulation across these major cultivated *Allium* species. A comparative view of *Allium* chromosome 2 is shown in Figure 2. The *Ms* locus conditioning restoration of male-fertility in S cytoplasm is the basis for most F_1_ hybrid production in onion, and has been mapped to this chromosome [7], and we observed association of markers in this region with seed yield from selfed F_2_, due to segregation at *Ms*, plants (McCallum et al., unpublished observations) in the 'W202A x Texas Grano 438' family used to map bulb composition QTL [9,10]. QTL have been reported in an adjacent chromosomal region for onion bulb composition [45] and *A. fistulosum* seedling vigor [13]. This comparative view allows ready comparison between the QTL locations and linked markers from these studies and provides potential markers for more detailed studies of these regions in these or other genetic backgrounds. Comparison of the onion and interspecific maps for this linkage group illustrates the typically good agreement between marker order and map length in these maps. The relatively small population sizes used to date in these studies do not yet allow conclusive identification of inversions or other major rearrangements in *Allium* maps.

## Conclusions

Previous comparative studies have shown no microsynteny of asparagus with rice or onion [46], suggesting that comparative genomic studies must focus within the genus *Allium*. AlliumMap provides an integrated point to access details of the genetic markers and sequence resources employed across multiple studies in cultivated *Allium*. New denser linkage maps and underlying marker resources currently under development using next-generation transcriptome sequencing will be deposited in AlliumMap in the near future and ongoing curation will ensure integration with past studies. Despite the rapid advances in sequencing technologies, the enormous size of *Allium* nuclear genomes will preclude full sequencing in the short term. However, reduced representation approaches are already practical and the data contained in AlliumMap will be valuable for aligning contigs from such studies with genetic and physical maps.

The resource will enable comparative genomics approaches, particularly for basic studies of plant physiology, metabolism and bioprotection in onion and *A. fistulosum*. Current transcriptome sequencing initiatives in onion will provide a rapidly expanding resource of anchor loci to expand the correspondences reported in this paper.

## Availability and requirements

The database and associated tools may be freely accessed at http://alliumgenetics.org. Data concerning AMAL assignments can be accessed as an RDF data sources at http://spreadsheets.google.com/pub?key=pUofr7CKURDMvUcUlAecgPQ&hl=en

## Abbreviations

AFLP, Amplified Fragment Length Polymorphism; AMAL, Alien monosomic addition line; EST, Expressed Sequence Tags; QTL, Quantitative Trait Loci; RFLP, Restriction fragment length polymorphism; SNP, Single-nucleotide polymorphism; SSR, Simple Sequence Repeat.

## Authors’ contributions

JM conceived the database and developed web interface. SB and JM curated mapping data and prepared manuscript YD configured CMAP and underlying database. MS curated data concerning monosomic alien addition lines. SvH constructed the interspecific *A. cepa* x *A. roylei* map. MPJ and FK conducted genetic marker evaluations in the *A. cepa* x *A. roylei* population.
